# [*N*′-(5-Bromo-2-oxidobenzyl­idene-κ*O*)-2-chloro­benzohydrazidato-κ^2^ *N*′,*O*](methanol-κ*O*)(methano­lato-κ*O*)oxido­vanadium(V)

**DOI:** 10.1107/S1600536811008774

**Published:** 2011-03-12

**Authors:** Fu-Ming Wang

**Affiliations:** a Department of Chemistry, Dezhou University, Dezhou Shandong 253023, People’s Republic of China

## Abstract

The V^V^ atom in the title complex, [V(C_14_H_8_BrClN_2_O_2_)(CH_3_O)O(CH_3_OH)], is six-coordinated by one phenolate O, one imine N and one enolic O atom of the hydrazone ligand, one oxide O atom, one methanol O atom and one methoxide O atom in a distorted octa­hedral geometry. The dihedral angle between the two benzene rings of the hydrazone ligand is 13.2 (3)°. The deviation of the V atom towards the oxide O atom from the plane defined by the three donor atoms of the hydrazone ligand and the meth­oxy O atom is 0.318 (2) Å. Bond lengths are comparable with those observed in similar oxidovanadium(V) complexes with hydrazone ligands. In the crystal, pairs of mol­ecules are linked through inter­molecular O—H⋯N hydrogen bonds, forming dimers.

## Related literature

For background to hydrazone compounds and their complexes, see: Seena *et al.* (2008 ▶); Bastos *et al.* (2008 ▶); Sarkar & Pal (2008 ▶); Nica *et al.* (2007 ▶). For similar oxidovanadium(V) complexes, see: Kurup *et al.* (2010 ▶); Rajak *et al.* (2000 ▶); Grüning *et al.* (1999 ▶); Mondal *et al.* (2009 ▶).
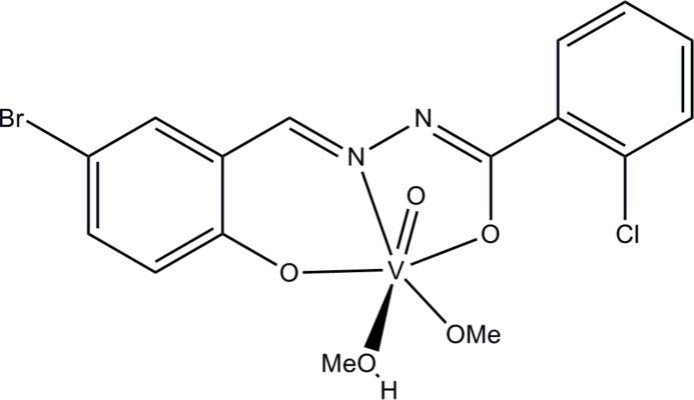


## Experimental

### 

#### Crystal data

[V(C_14_H_8_BrClN_2_O_2_)(CH_3_O)O(CH_4_O)]
*M* *_r_* = 481.60
Monoclinic, 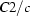

*a* = 28.09 (2) Å
*b* = 7.992 (6) Å
*c* = 20.163 (14) Å
β = 121.854 (7)°
*V* = 3844 (5) Å^3^
*Z* = 8
Mo *K*α radiation
μ = 2.76 mm^−1^
*T* = 298 K
0.30 × 0.27 × 0.23 mm

#### Data collection

Bruker SMART CCD area-detector diffractometer
Absorption correction: multi-scan (*SADABS*; Sheldrick, 1996 ▶) *T*_min_ = 0.491, *T*_max_ = 0.569
9750 measured reflections
4081 independent reflections
2266 reflections with *I* > 2σ(*I*)
*R*_int_ = 0.056

#### Refinement

*R*[*F* ^2^ > 2σ(*F* ^2^)] = 0.045
*wR*(*F* ^2^) = 0.103
*S* = 1.02
4081 reflections
240 parameters
1 restraint
H atoms treated by a mixture of independent and constrained refinement
Δρ_max_ = 0.45 e Å^−3^
Δρ_min_ = −0.44 e Å^−3^

### 

Data collection: *SMART* (Bruker, 1998 ▶); cell refinement: *SAINT* (Bruker, 1998 ▶); data reduction: *SAINT*; program(s) used to solve structure: *SHELXS97* (Sheldrick, 2008 ▶); program(s) used to refine structure: *SHELXL97* (Sheldrick, 2008 ▶); molecular graphics: *SHELXTL* (Sheldrick, 2008 ▶); software used to prepare material for publication: *SHELXTL*.

## Supplementary Material

Crystal structure: contains datablocks global, I. DOI: 10.1107/S1600536811008774/qm2003sup1.cif

Structure factors: contains datablocks I. DOI: 10.1107/S1600536811008774/qm2003Isup2.hkl

Additional supplementary materials:  crystallographic information; 3D view; checkCIF report

## Figures and Tables

**Table 1:** Selected bond lengths (Å)

V1—O4	1.582 (3)
V1—O3	1.765 (3)
V1—O1	1.859 (3)
V1—O2	1.957 (3)
V1—N1	2.134 (3)
V1—O5	2.403 (4)

**Table 2:** Hydrogen-bond geometry (Å, °)

*D*—H⋯*A*	*D*—H	H⋯*A*	*D*⋯*A*	*D*—H⋯*A*
O5—H5⋯N2^i^	0.85 (4)	2.06 (4)	2.906 (4)	178 (5)

Symmetry code: (i) 
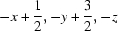
.

**Fig. 1.**
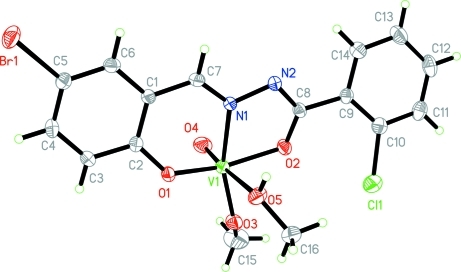
The asymmetric unit of the title compound, showing the atom-numbering scheme. Displacement ellipsoids are drawn at the 30% probability level.

**Fig. 2.**
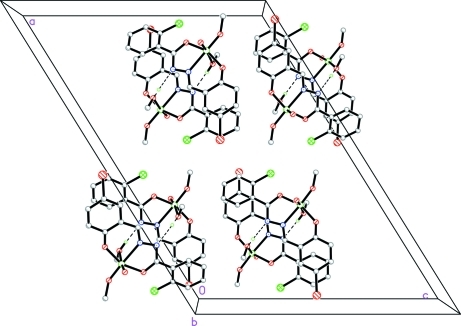
The molecular packing of the title complex, viewed along the b axis.

## supplementary crystallographic information

### Comment

Hydrazone compounds and their oxovanadium complexes have received much attention due to their structures and biological properties (Seena *et al.*, 2008; Bastos *et al.*, 2008; Sarkar & Pal, 2008; Nica *et al.*, 2007). In this paper, the title new oxovanadium(V) complex with a hydrazone ligand is reported.

The V^V^ atom in the title complex, Fig. 1, is six-coordinated by one phenolic O, one imine N, and one enolic O atoms of the hydrazone ligand, by one oxo O atom, and by two O atoms respectively from a methanol molecule and a methoxide ligand, forming a distorted octahedral geometry. The dihedral angle between the two benzene rings of the hydrazone ligand is 13.2 (3)°. The deviation of the V atom from the plane defined by the three donor atoms of the hydrazone ligand and the methoxy O atom towards the oxo O atom is 0.318 (2) Å. The coordinate bond lengths and angles (Table 1) are comparable with those observed in similar oxovanadium(V) complexes (Kurup *et al.*, 2010; Rajak *et al.*, 2000; Grüning *et al.*, 1999; Mondal *et al.*, 2009). In the crystal structure, adjacent two molecules are linked through intermolecular O—H···N hydrogen bonds (Table 2), to form a dimer, as shown in Fig. 2.

### Experimental

5-Bromosalicylaldehyde (1 mmol, 0.20 g), 2-chlorobenzohydrazide 1 mmol, 0.17 g), and VO(acac)_2_ (1 mmol, 0.26 g) were mixed in methanol (30 ml). The mixture was boiled under reflux for 2 h, then cooled to room temperature. Brown block-like single crystals, suitable for X-ray diffraction, were formed after slow evaporation of the solution in air for a few days.

### Refinement

H5 atom was located from a difference Fourier map and refined isotropically. The O5—H5 distance is restrained to 0.85 (1) Å. The remaining hydrogen atoms were placed in geometrically idealized positions and constrained to ride on their parent atoms, with C—H distances of 0.93–0.96 Å, and with *U*_iso_(H) set at 1.2*U*_eq_(C) and 1.5*U*_eq_(C_methyl_).

### Crystal data

**Table:**

[V(C_14_H_8_BrClN_2_O_2_)(CH_3_O)O(CH_4_O)]	*F*(000) = 1920
*M**_r_* = 481.60	*D*_x_ = 1.664 Mg m^−^^3^
Monoclinic, *C*2/*c*	Mo *K*α radiation, λ = 0.71073 Å
*a* = 28.09 (2) Å	Cell parameters from 1798 reflections
*b* = 7.992 (6) Å	θ = 2.3–25.0°
*c* = 20.163 (14) Å	µ = 2.76 mm^−^^1^
β = 121.854 (7)°	*T* = 298 K
*V* = 3844 (5) Å^3^	Block, brown
*Z* = 8	0.30 × 0.27 × 0.23 mm

### Data collection

**Table:**

Bruker SMART CCD area-detector diffractometer	4081 independent reflections
Radiation source: fine-focus sealed tube	2266 reflections with *I* > 2σ(*I*)
graphite	*R*_int_ = 0.056
ω scans	θ_max_ = 27.0°, θ_min_ = 2.4°
Absorption correction: multi-scan (*SADABS*; Sheldrick, 1996)	*h* = −28→35
*T*_min_ = 0.491, *T*_max_ = 0.569	*k* = −9→9
9750 measured reflections	*l* = −25→25

### Refinement

**Table:**

Refinement on *F*^2^	Primary atom site location: structure-invariant direct methods
Least-squares matrix: full	Secondary atom site location: difference Fourier map
*R*[*F*^2^ > 2σ(*F*^2^)] = 0.045	Hydrogen site location: inferred from neighbouring sites
*wR*(*F*^2^) = 0.103	H atoms treated by a mixture of independent and constrained refinement
*S* = 1.02	*w* = 1/[σ^2^(*F*_o_^2^) + (0.0359*P*)^2^] where *P* = (*F*_o_^2^ + 2*F*_c_^2^)/3
4081 reflections	(Δ/σ)_max_ < 0.001
240 parameters	Δρ_max_ = 0.45 e Å^−^^3^
1 restraint	Δρ_min_ = −0.44 e Å^−^^3^

### Special details

**Table:**

Geometry. All esds (except the esd in the dihedral angle between two l.s. planes) are estimated using the full covariance matrix. The cell esds are taken into account individually in the estimation of esds in distances, angles and torsion angles; correlations between esds in cell parameters are only used when they are defined by crystal symmetry. An approximate (isotropic) treatment of cell esds is used for estimating esds involving l.s. planes.
Refinement. Refinement of F^2^ against ALL reflections. The weighted R-factor wR and goodness of fit S are based on F^2^, conventional R-factors R are based on F, with F set to zero for negative F^2^. The threshold expression of F^2^ > 2sigma(F^2^) is used only for calculating R-factors(gt) etc. and is not relevant to the choice of reflections for refinement. R-factors based on F^2^ are statistically about twice as large as those based on F, and R- factors based on ALL data will be even larger.

### Fractional atomic coordinates and isotropic or equivalent isotropic displacement parameters (Å^2^)

**Table:**

	*x*	*y*	*z*	*U*_iso_*/*U*_eq_	
V1	0.33992 (3)	0.48966 (10)	0.15964 (4)	0.0355 (2)	
Br1	0.05406 (2)	0.13036 (7)	0.00218 (3)	0.05439 (19)	
Cl1	0.44356 (6)	0.87745 (17)	0.12452 (7)	0.0663 (4)	
H5	0.2799 (16)	0.809 (6)	0.1145 (18)	0.099*	
N1	0.27232 (13)	0.5095 (4)	0.04108 (16)	0.0281 (8)	
N2	0.28364 (14)	0.5947 (4)	−0.01011 (18)	0.0306 (8)	
O1	0.28365 (12)	0.4401 (4)	0.17896 (15)	0.0428 (8)	
O2	0.37010 (11)	0.6037 (3)	0.10369 (15)	0.0392 (8)	
O3	0.39495 (11)	0.5587 (4)	0.25184 (14)	0.0418 (8)	
O4	0.35806 (13)	0.3060 (4)	0.15302 (15)	0.0519 (9)	
O5	0.30672 (12)	0.7684 (4)	0.15650 (16)	0.0441 (8)	
C1	0.20329 (17)	0.3624 (5)	0.0557 (2)	0.0298 (10)	
C2	0.23468 (17)	0.3619 (5)	0.1384 (2)	0.0336 (10)	
C3	0.21228 (18)	0.2811 (6)	0.1779 (2)	0.0425 (12)	
H3	0.2332	0.2758	0.2321	0.051*	
C4	0.15989 (18)	0.2100 (6)	0.1374 (2)	0.0422 (12)	
H4	0.1456	0.1574	0.1644	0.051*	
C5	0.12819 (16)	0.2159 (5)	0.0566 (2)	0.0351 (11)	
C6	0.14949 (17)	0.2896 (5)	0.0161 (2)	0.0346 (11)	
H6	0.1281	0.2914	−0.0382	0.042*	
C7	0.22241 (18)	0.4472 (5)	0.0110 (2)	0.0331 (10)	
H7	0.1975	0.4580	−0.0426	0.040*	
C8	0.33695 (17)	0.6358 (5)	0.0297 (2)	0.0309 (10)	
C9	0.36154 (17)	0.7211 (5)	−0.0115 (2)	0.0310 (10)	
C10	0.40956 (18)	0.8227 (5)	0.0260 (2)	0.0393 (11)	
C11	0.43223 (19)	0.8885 (6)	−0.0144 (3)	0.0482 (13)	
H11	0.4648	0.9521	0.0118	0.058*	
C12	0.4068 (2)	0.8607 (6)	−0.0937 (3)	0.0546 (14)	
H12	0.4218	0.9076	−0.1210	0.065*	
C13	0.3589 (2)	0.7628 (6)	−0.1326 (3)	0.0530 (13)	
H13	0.3420	0.7419	−0.1857	0.064*	
C14	0.33694 (17)	0.6971 (5)	−0.0914 (2)	0.0393 (11)	
H14	0.3043	0.6340	−0.1180	0.047*	
C15	0.44932 (19)	0.4960 (8)	0.3020 (3)	0.0801 (19)	
H15A	0.4502	0.3791	0.2918	0.120*	
H15B	0.4597	0.5109	0.3552	0.120*	
H15C	0.4752	0.5552	0.2932	0.120*	
C16	0.3407 (2)	0.9086 (6)	0.1983 (3)	0.0563 (14)	
H16A	0.3700	0.8736	0.2494	0.084*	
H16B	0.3181	0.9920	0.2030	0.084*	
H16C	0.3569	0.9547	0.1706	0.084*	

### Atomic displacement parameters (Å^2^)

**Table:**

	*U*^11^	*U*^22^	*U*^33^	*U*^12^	*U*^13^	*U*^23^
V1	0.0370 (5)	0.0392 (5)	0.0266 (4)	−0.0050 (4)	0.0141 (4)	0.0019 (4)
Br1	0.0345 (3)	0.0605 (4)	0.0636 (3)	−0.0119 (3)	0.0227 (3)	−0.0005 (3)
Cl1	0.0644 (9)	0.0763 (10)	0.0548 (8)	−0.0275 (8)	0.0292 (7)	−0.0141 (7)
N1	0.032 (2)	0.028 (2)	0.0254 (17)	−0.0033 (17)	0.0157 (16)	0.0003 (16)
N2	0.033 (2)	0.032 (2)	0.0292 (18)	−0.0039 (17)	0.0180 (17)	0.0002 (16)
O1	0.0400 (19)	0.058 (2)	0.0281 (15)	−0.0200 (16)	0.0165 (15)	−0.0009 (15)
O2	0.0330 (17)	0.049 (2)	0.0303 (16)	−0.0054 (15)	0.0130 (14)	0.0083 (14)
O3	0.0318 (18)	0.055 (2)	0.0286 (16)	0.0001 (16)	0.0094 (15)	0.0063 (15)
O4	0.067 (2)	0.042 (2)	0.0389 (18)	0.0046 (18)	0.0226 (17)	0.0038 (15)
O5	0.041 (2)	0.038 (2)	0.0410 (18)	−0.0035 (17)	0.0129 (15)	−0.0027 (16)
C1	0.033 (2)	0.028 (3)	0.028 (2)	−0.001 (2)	0.015 (2)	0.0011 (19)
C2	0.033 (3)	0.034 (3)	0.034 (2)	−0.005 (2)	0.018 (2)	0.000 (2)
C3	0.047 (3)	0.053 (3)	0.032 (2)	−0.012 (3)	0.024 (2)	−0.003 (2)
C4	0.045 (3)	0.045 (3)	0.047 (3)	−0.010 (2)	0.031 (3)	0.001 (2)
C5	0.031 (3)	0.036 (3)	0.038 (2)	−0.002 (2)	0.018 (2)	−0.001 (2)
C6	0.037 (3)	0.033 (3)	0.028 (2)	0.000 (2)	0.013 (2)	0.000 (2)
C7	0.034 (3)	0.037 (3)	0.025 (2)	−0.001 (2)	0.013 (2)	0.000 (2)
C8	0.035 (3)	0.031 (3)	0.033 (2)	0.001 (2)	0.022 (2)	0.002 (2)
C9	0.033 (2)	0.025 (3)	0.037 (2)	0.003 (2)	0.019 (2)	0.002 (2)
C10	0.040 (3)	0.034 (3)	0.043 (3)	0.005 (2)	0.021 (2)	0.004 (2)
C11	0.038 (3)	0.048 (3)	0.064 (3)	−0.009 (2)	0.031 (3)	0.000 (3)
C12	0.060 (3)	0.057 (4)	0.070 (4)	0.002 (3)	0.050 (3)	0.015 (3)
C13	0.054 (3)	0.069 (4)	0.046 (3)	0.001 (3)	0.033 (3)	0.009 (3)
C14	0.032 (3)	0.048 (3)	0.039 (3)	0.005 (2)	0.020 (2)	0.004 (2)
C15	0.039 (3)	0.116 (5)	0.058 (3)	0.015 (4)	0.007 (3)	0.007 (4)
C16	0.066 (4)	0.053 (4)	0.043 (3)	−0.013 (3)	0.024 (3)	−0.007 (3)

### Geometric parameters (Å, °)

**Table:**

V1—O4	1.582 (3)	C4—C5	1.384 (5)
V1—O3	1.765 (3)	C4—H4	0.9300
V1—O1	1.859 (3)	C5—C6	1.374 (5)
V1—O2	1.957 (3)	C6—H6	0.9300
V1—N1	2.134 (3)	C7—H7	0.9300
V1—O5	2.403 (4)	C8—C9	1.496 (5)
Br1—C5	1.896 (4)	C9—C14	1.390 (5)
Cl1—C10	1.746 (4)	C9—C10	1.405 (6)
N1—C7	1.298 (5)	C10—C11	1.375 (6)
N1—N2	1.406 (4)	C11—C12	1.383 (6)
N2—C8	1.314 (5)	C11—H11	0.9300
O1—C2	1.328 (5)	C12—C13	1.386 (6)
O2—C8	1.301 (4)	C12—H12	0.9300
O3—C15	1.406 (5)	C13—C14	1.375 (5)
O5—C16	1.424 (5)	C13—H13	0.9300
O5—H5	0.85 (4)	C14—H14	0.9300
C1—C6	1.409 (5)	C15—H15A	0.9600
C1—C2	1.418 (5)	C15—H15B	0.9600
C1—C7	1.439 (5)	C15—H15C	0.9600
C2—C3	1.404 (5)	C16—H16A	0.9600
C3—C4	1.374 (5)	C16—H16B	0.9600
C3—H3	0.9300	C16—H16C	0.9600
			
O4—V1—O3	103.70 (15)	C5—C6—C1	120.9 (4)
O4—V1—O1	99.61 (15)	C5—C6—H6	119.6
O3—V1—O1	102.39 (13)	C1—C6—H6	119.6
O4—V1—O2	97.29 (14)	N1—C7—C1	123.8 (4)
O3—V1—O2	93.25 (13)	N1—C7—H7	118.1
O1—V1—O2	153.46 (12)	C1—C7—H7	118.1
O4—V1—N1	96.33 (13)	O2—C8—N2	123.2 (3)
O3—V1—N1	157.41 (14)	O2—C8—C9	117.8 (4)
O1—V1—N1	84.17 (13)	N2—C8—C9	119.1 (4)
O2—V1—N1	73.72 (13)	C14—C9—C10	116.9 (4)
O4—V1—O5	174.59 (12)	C14—C9—C8	119.2 (4)
O3—V1—O5	81.36 (12)	C10—C9—C8	123.9 (4)
O1—V1—O5	80.92 (13)	C11—C10—C9	121.1 (4)
O2—V1—O5	80.39 (12)	C11—C10—Cl1	115.6 (4)
N1—V1—O5	78.35 (11)	C9—C10—Cl1	123.4 (3)
C7—N1—N2	116.7 (3)	C10—C11—C12	120.4 (4)
C7—N1—V1	126.8 (3)	C10—C11—H11	119.8
N2—N1—V1	116.5 (2)	C12—C11—H11	119.8
C8—N2—N1	107.4 (3)	C11—C12—C13	119.9 (4)
C2—O1—V1	133.7 (3)	C11—C12—H12	120.0
C8—O2—V1	119.2 (2)	C13—C12—H12	120.0
C15—O3—V1	131.4 (3)	C14—C13—C12	119.0 (4)
C16—O5—V1	125.8 (3)	C14—C13—H13	120.5
C16—O5—H5	105 (4)	C12—C13—H13	120.5
V1—O5—H5	121 (4)	C13—C14—C9	122.7 (4)
C6—C1—C2	118.7 (4)	C13—C14—H14	118.7
C6—C1—C7	118.8 (3)	C9—C14—H14	118.7
C2—C1—C7	122.2 (4)	O3—C15—H15A	109.5
O1—C2—C3	119.7 (4)	O3—C15—H15B	109.5
O1—C2—C1	121.4 (3)	H15A—C15—H15B	109.5
C3—C2—C1	118.8 (4)	O3—C15—H15C	109.5
C4—C3—C2	120.9 (4)	H15A—C15—H15C	109.5
C4—C3—H3	119.6	H15B—C15—H15C	109.5
C2—C3—H3	119.6	O5—C16—H16A	109.5
C3—C4—C5	120.5 (4)	O5—C16—H16B	109.5
C3—C4—H4	119.8	H16A—C16—H16B	109.5
C5—C4—H4	119.8	O5—C16—H16C	109.5
C6—C5—C4	120.2 (4)	H16A—C16—H16C	109.5
C6—C5—Br1	120.0 (3)	H16B—C16—H16C	109.5
C4—C5—Br1	119.8 (3)		

### Hydrogen-bond geometry (Å, °)

**Table:**

*D*—H···*A*	*D*—H	H···*A*	*D*···*A*	*D*—H···*A*
O5—H5···N2^i^	0.85 (4)	2.06 (4)	2.906 (4)	178 (5)

Symmetry codes: (i) −*x*+1/2, −*y*+3/2, −*z*.
